# Observations on an Extraordinary Disease, Followed by the Death of the Person, and Occasioned by the Presence of an Insect Found Living in the Substance of the Liver

**Published:** 1803-02-01

**Authors:** Deleau Desfontaines

**Affiliations:** St. Germain, and read at the Medical Society, 12th Prairial, 10th Year


*Observations on an extraordinary Disease, followed by the Death of the Person, and occasioned by the Presence of an Insect found living in the Substance of the Liver; by* Cit. DELEAU DESFONTAINES, M. D. *of St. Germain, and read at the Medical Society, 12th Prairial, 10th Year.*

CIT. DESFONTAINES informs us, that he was desired to see a man, aged 33, who had been previously strong and robust, his temperament sanguineo-melancholic. On first seeing him, he complained of sharp pains in his stomach, and difficult digestion, which was now and then relieved by a slight diarrhœa; the colour of his skin was pale and livid; crude urine sometimes tinged yellow, and charged with a glairy sediment; tongue foul, and the pulse small and hard. These symptoms led the author to suspect visceral obstruction; he ordered him diluents, such as whey, veal broth, and an infusion of plants of the checoranous kind; and in a few days the patient was purged, which seemed to serve him. After the lapse of three weeks the patient complained of vertigo; the pains in the head became habitual, and those of the stomach more severe, and extended down to the great lobe of the liver; the patient lost all appetite for food, and he frequently fainted, during which time the skin became of the colour of saffron; a fœtid smell issued from the mouth; the urine became thick and of a yellowish brown, and this kind of crisis terminated by a discharge of bile. The author acknowledges the uncertainty he felt as to the nature of the disease, the diagnostic of which he found difficult to determine; while, on the one hand, he had reason to conjecture the disease of some of the gieat viscera, particularly the liver, he dreaded, on the other, the existence of worms, or the presence of biliary calculi. After having recourse to laxatives, bitters, injections per anum, and anodynes, he ordered the application of an emollient cataplasm with some drops of laudanum to the belly, with the intention of diminishing the excessive pain; five weeks had elapsed since his first visit, all the symptoms were aggravated, the stomach rejected food, the fever increased, and the extremities became œdematous. He determined to administer half a drachm of vermifuge powder, in the composition of which calomel entered, without producing any sensible effect. The following day he repeated the dose, which was followed by an evacuation of blood that terminated the existence of the patient. Leave was granted to examine the body.

The

The contents of the thorax offered nothing remarkable; the stomach was contracted, and its superior orifice folded like a purse; its interior membrane was red and inflamed, and shewed some gangrenous spots; the small intestines were swelled and distended with air; the pancreas was larger than usual; the gall bladder was empty and much smaller than its ordinary size, and contained two small calculi; the liver was much diminished from its usual volume, and was in many places hard and schirrous, in others pale and livid; towards the middle of the concave surface of the great lobe was perceived a kind of cavity six or seven lines in diameter, and four or five in depth, filled with a black thick humour, and out of which came an insect yet alive; it was a worm of an extraordinary kind, and resembling no way those which practitioners have described as hepatic worms. It was 4 inches long, and of the thickness of a silk worm of the largest size; its colour was of a brownish red, and its body was articulated in the form of rings; each was marked by a white spot, in the middle of which was implanted a hair of a resisting nature, and extremely sharp, and which, seen through a lens, resembled the quills of a porcupine; the head of the insect was armed with a kind of proboscis also articulated; the inferior extremity was terminated by a large flat tail, similar to that of a crab. Cit. Desfontaines concludes by observing, that whether this reptile was produced or engendered in the substance of the liver, or introduced already formed into the body, he does not pretend to say; he only pretends to establish the fact of its existence, and to shew that its presence in the liver was the original cause of the disease. This observation is followed by some remarks on the case by Cit. Double, M. D. and member of the Society; in which he enters into a detail of the symptoms which attend on, and distinguish between schirrous liver and the presence of calculi in the gall bladder, and of which he draws proofs from Morgagni, Bianchi, &c. and he seems to think that the original cause of disease in this case, was the calculi found, and not the insect. We think it highly probable that the existence of this being might more probably be a consequence of a previous derangement. There are many reflections that will strike the intelligent reader on the perusal of the history, as well as the treatment of this case, but which we forbear to observe. It is to be lamented that Cit. Desfontaines did not present the insect to the society. The presence of worms in the liver is attested by Bianchi, Glisson, &c. and a case is also mentioned in the London Medical Observations.

Copy

